# Optimizing multi-objective task scheduling in fog computing with GA-PSO algorithm for big data application

**DOI:** 10.3389/fdata.2024.1358486

**Published:** 2024-02-21

**Authors:** Muhammad Saad, Rabia Noor Enam, Rehan Qureshi

**Affiliations:** ^1^Computer Engineering Department, Sir Syed University of Engineering and Technology, Karachi, Pakistan; ^2^Software Engineering Department, Sir Syed University of Engineering and Technology, Karachi, Pakistan; ^3^University of Southern Queensland, Springfield, QLS, Australia

**Keywords:** fog computing, task scheduling, genetic algorithm, particle swarm optimization, hybrid algorithm, hybrid GA-PSO, fog computing (FC), cloud computing

## Abstract

As the volume and velocity of Big Data continue to grow, traditional cloud computing approaches struggle to meet the demands of real-time processing and low latency. Fog computing, with its distributed network of edge devices, emerges as a compelling solution. However, efficient task scheduling in fog computing remains a challenge due to its inherently multi-objective nature, balancing factors like execution time, response time, and resource utilization. This paper proposes a hybrid Genetic Algorithm (GA)-Particle Swarm Optimization (PSO) algorithm to optimize multi-objective task scheduling in fog computing environments. The hybrid approach combines the strengths of GA and PSO, achieving effective exploration and exploitation of the search space, leading to improved performance compared to traditional single-algorithm approaches. The proposed hybrid algorithm results improved the execution time by 85.68% when compared with GA algorithm, by 84% when compared with Hybrid PWOA and by 51.03% when compared with PSO algorithm as well as it improved the response time by 67.28% when compared with GA algorithm, by 54.24% when compared with Hybrid PWOA and by 75.40% when compared with PSO algorithm as well as it improved the completion time by 68.69% when compared with GA algorithm, by 98.91% when compared with Hybrid PWOA and by 75.90% when compared with PSO algorithm when various tasks inputs are given. The proposed hybrid algorithm results also improved the execution time by 84.87% when compared with GA algorithm, by 88.64% when compared with Hybrid PWOA and by 85.07% when compared with PSO algorithm it improved the response time by 65.92% when compared with GA algorithm, by 80.51% when compared with Hybrid PWOA and by 85.26% when compared with PSO algorithm as well as it improved the completion time by 67.60% when compared with GA algorithm, by 81.34% when compared with Hybrid PWOA and by 85.23% when compared with PSO algorithm when various fog nodes are given.

## 1 Introduction

### 1.1 A new era of big data and its impact on computing

The pace of data generation is increasing rapidly due to the widespread use of IoT sensors and devices, as well as people's increasing reliance on online services and social media (Chandra and Verma, 2023; Mortaheb and Jankowski, 2023). The phenomenon known as Big Data has had a significant impact on several aspects of modern life, such as healthcare, finance, and transportation (Mohanty, 2015; Fanelli et al., 2023).

### 1.2 Challenges and opportunities of big data

There are substantial obstacles to overcome, despite the fact that Big Data has enormous potential for insight and innovation:

**Data Volume**: Innovative storage methods and scalable computer infrastructure are necessary for managing and storing huge datasets (Kim et al., 2023; Wang and Yin, 2023).

**Data Velocity**: In order to derive useful insights from data streams in real-time as they are being created, high-performance computing skills are required (Bharany, 2023; Olawoyin et al., 2023).

**Data Variety**: Flexible processing methods and sophisticated analytics approaches are required due to the different nature of data sources, which might range from structured databases to unstructured social media postings (Khang et al., 2023; Qi et al., 2023).

### 1.3 The emergence of fog computing: a paradigm shift in data processing

Big Data is highly challenging for the conventional cloud computing architecture, which relies on centralizing data and processing it in distant data centers, to effectively manage (Rosati et al., 2023; Sheng et al., 2023). A unique method to data processing is necessary because of the excessive latency, poor scalability, and security concerns that have been raised against it. At this time, fog computing, which is a paradigm switcher, comes into being.

Computing in the fog pushes the computational capabilities of the cloud closer to the edge of the network (Bebortta et al., 2023; Hornik et al., 2023). Fog computing is used when data is produced and consumed at the network's peripheral. The ability of fog computing to process data locally on a network of devices that have restricted capabilities (fog nodes) is one of the many benefits that fog computing provides. Some of these advantages includes the amount of latency that an application experiences is significantly reduced when it processes data locally as opposed to forwarding it to faraway cloud servers (Hussein et al., 2023). This is because the amount of delay that an application experiences is reduced greatly. This is a very important piece of information, particularly for real-time applications that need rapid replies and opportunities for decision. The Internet of Things (IoT) is able to effectively handle enormous amounts of data and expand without encountering any challenges as the network grows. This is made possible by distributed processing, which is made possible by fog computing (Fazel et al., 2023).

The processing of sensitive data locally decreases the quantity of data that is transferred to distant servers, which in turn promotes both security and privacy (Raj, 2023). This results in enhanced security. Furthermore, this is particularly helpful for applications that deal with information that is of crucial importance.

Offloading workloads to fog nodes may help you decrease the operating expenses that are connected with cloud infrastructure and maximize the utilization of other resources (Das and Inuwa, 2023; Mohamed et al., 2023). When you do this, you can also lower the costs associated with cloud infrastructure.

### 1.4 Multi-objective task scheduling: orchestrating tasks in the fog with big data considerations

Despite the fact that fog computing offers a number of benefits for the processing of large amounts of data, one of the most significant challenges is still the discovery of an efficient method to schedule operations within the network. This involves allocating work to appropriate fog nodes, taking into consideration the many objectives that are being pursued is Minimizing Execution Time. Rapid response and customer satisfaction are of the utmost importance in real-time applications due to the potentially disastrous effects of delays. Finding the most efficient way to do things is the key to achieving this goal.

Customers anticipate prompt responses while interacting with apps. Making the most efficient use of time is crucial. Particularly in interactive situations, minimizing reaction time is crucial for ensuring a satisfying user experience and avoiding disappointment. An important factor in assessing the system's overall efficacy and efficiency is the sum of all job completion times. Reducing the completion time is of the utmost importance. Finding the sweet spot for job completion times is critical for avoiding network bottlenecks and ensuring effective task processing.

By reducing the amount of data transported over unsecure networks, processing data near to its origin helps to alleviate network congestion and enhances data privacy. To do this, data is used to its fullest extent when it is located close to its point of origin. The vast majority of the time, these goals are diametrically opposed. A job that is prioritized based on execution time may see an increase in response time owing to network congestion or queueing if it is assigned to a strong fog node. The purpose of this is to show how the execution time may be prioritized. Finding the sweet spot for task schedule optimization inside the Big Data framework requires a multi-objective strategy. Using this approach well requires thinking about all of these goals at once and finding the best middle ground.

**Figure 1:**
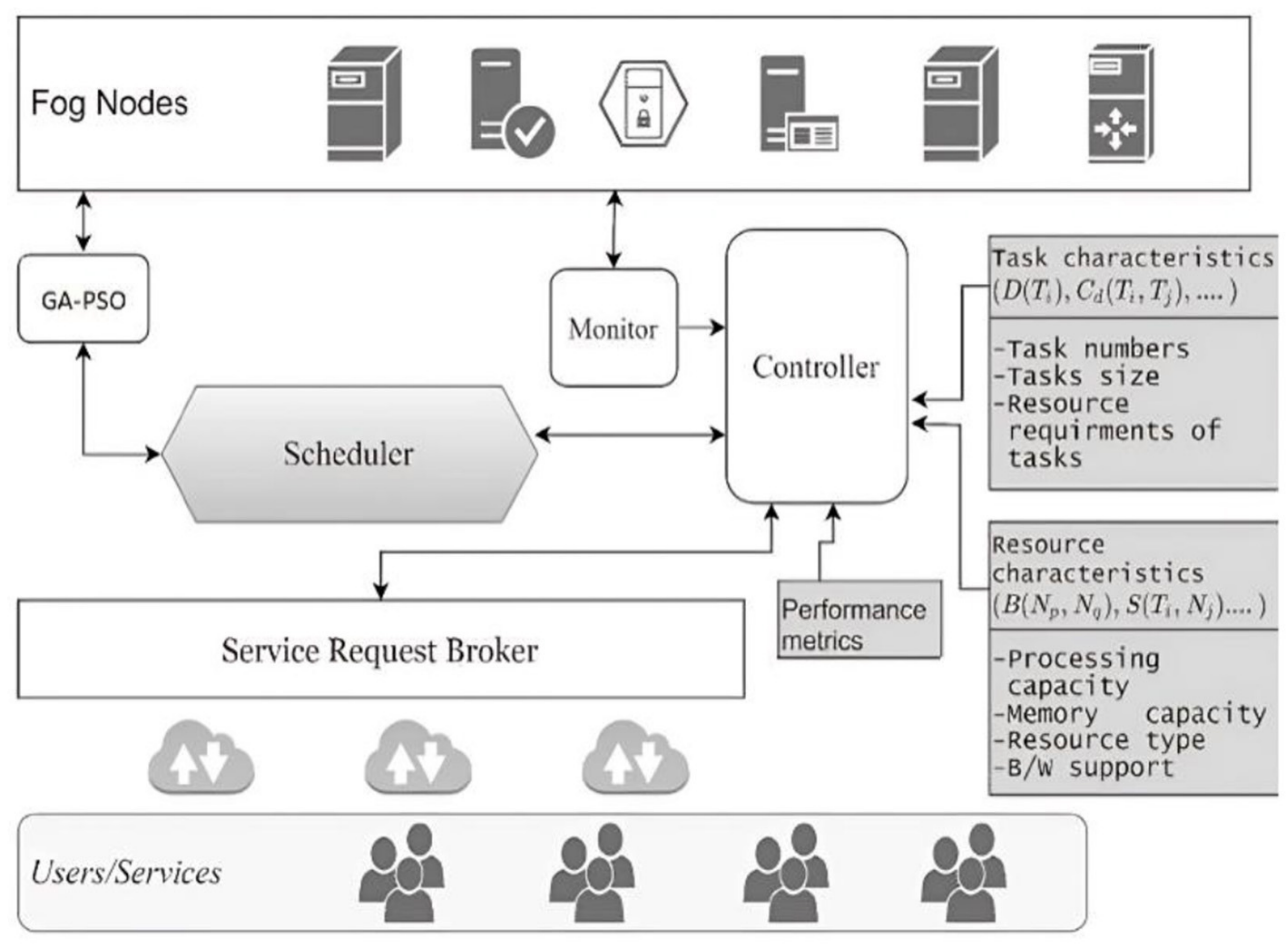
The task scheduling architecture in the fog environment.

For efficient Big Data management in fog, a hybrid GA-PSO algorithm seems to be a viable option. This is because it overcomes the shortcomings of previous methods and effectively deals with Big Data. Effective multi-objective task scheduling in fog computing settings is the goal of this approach, which combines the strengths of genetic algorithm (GA) with particle swarm optimization (PSO). The two methods work together to achieve this goal. The fog computing environment's task scheduling mechanism is shown in Figure 1.

This study aims to enhance task scheduling in fog computing for big data applications, motivated by the need to overcome resource constraints in fog settings while meeting the rigorous demands of large data processing. We provide a hybrid GA-PSO method that utilizes the collective advantages of global exploration and rapid convergence to effectively manage the inherent trade-offs in multi-objective optimization. Our objective is to explore the fundamental element of fog computing in order to fully use its potential in various applications, improve user satisfaction, and optimize energy consumption, eventually creating a more robust and environmentally friendly large data processing environment. The remaining portions of the article are divided into the following sections for your convenience: In Section 2, we will conduct a review of the most current research that has been conducted in this area. In the third section, the structure of the algorithm that is used to schedule jobs as well as its definitions are discussed. The performance assessment of the multi-objective task scheduling problem in a fog environment is discussed in Section 4, which has a wealth of information on the subject. In addition to the data obtained from the experiment, this section also includes a summary of the results obtained from the experiment. In Section 5, a synopsis of the work that will be carried out in the future is offered.

## 2 Literature review

Several researchers have proposed various approaches to address the multi-objective task scheduling problem in fog computing for Big Data applications. These approaches can be broadly categorized as follows:

### 2.1 Heuristic-based algorithms

These rely on domain-specific knowledge and heuristics to assign tasks to fog nodes. Simple and easy to implement, but often struggle to adapt to diverse scenarios and may not find optimal solutions. Examples: Greedy algorithm, First-Come First-Serve (FCFS).

### 2.2 Single-objective optimization algorithms

These focus on optimizing only one objective, typically execution time or resource utilization. May not consider other important objectives and may not lead to optimal solutions when considering multiple conflicting objectives. Examples:

Shortest Job First (SJF), Round-Robin scheduling.

### 2.3 Metaheuristic algorithms

These are population-based optimization algorithms inspired by natural phenomena. Can handle complex search spaces and explore diverse solutions effectively. Examples: Genetic Algorithm (GA), Particle Swarm Optimization (PSO), Ant Colony Optimization (ACO).

### 2.4 Hybrid algorithms

Combine the strengths of different optimization algorithms to achieve better performance. Can leverage the exploration capabilities of GA and the exploitation strengths of PSO for a more balanced and comprehensive search. Examples: PSOWOA.

Here we provide a literature review of job scheduling algorithms that make use of one of these methods. A Multi-Objectives Grey Wolf Optimizer (MGWO) algorithm hosted on the fog broker to optimize both delay and energy consumption was presented by Saif et al. (2023). Simulation results show MGWO's effectiveness compared to existing algorithms in reducing both objectives.

Combining fog computing with cloud computing allows processing near data production sites for faster speeds and reduced bandwidth needs, especially beneficial for real-time IoT applications. However, limited resources in fog nodes necessitate efficient task scheduling to meet dynamic demands. This survey analyzes existing techniques, categorized into machine learning, heuristic, metaheuristic, and deterministic approaches, evaluating them based on execution time, resource utilization, and various other parameters is presented by Hosseinzadeh et al. (2023). It reveals that metaheuristic-based methods are most common (38%), followed by heuristic (30%), machine learning (23%), and deterministic (9%). Energy consumption is the most addressed objective (19%). A number of future options for enhancing fog computing job scheduling are highlighted in the conclusion of the paper. These future avenues include responding to dynamic situations, adding security and privacy, and leveraging improvements in artificial intelligence and machine learning applications.

An approach known as a hybrid meta-heuristic optimization algorithm (HMOA) was published by Jakwa et al. (2023) for the purpose of scheduling tasks in fog computing in an energy-efficient manner. HMOA combines MPSO with deterministic spanning tree to overcome the drawbacks of separate methods. The hybrid MPSO-SPT allocates and manages resources, while MPSO schedules user tasks across fog devices. HMOA uses resources and energy better than state-of-the-art algorithms. The usage and evaluation of iFogSim permitted this. Future hybrid experiments will investigate execution time. This article addresses multi-objective scheduling in fog computing, where remote resources and unexpected demands are prevalent. Dai et al. (2023) design a multi-objective optimization model that maximizes time delay and energy use after proposing a dynamic priority adjustment methodology for task offloading. A model that optimizes priority adjustment follows. Due to its ability to manage complicated Pareto fronts and reduce reaction time and energy consumption, the M-E-AWA algorithm (MOEA/D with adaptive weight adjustment) may be useful for fog task scheduling.

The goal of this project is to schedule scientific activities in fog computing while conserving energy. HDSOS-GOA is a new hybrid approach proposed in this study. Symbiotic Organisms Search (SOS) and Grasshopper Optimization Algorithm (GOA) algorithms are used with learning automata for dynamic selection. Mohammadzadeh et al. (2023) use the HEFT heuristic and DVFS methodology to optimize energy usage and job scheduling. Provided this way HDSOS-GOA surpasses rival scheduling algorithms in energy use, makespan, and completion time, according to extensive testing. Thus, it may be a low-power fog computing option.

To efficiently manage requests from the Internet of Things (IoT) in a cloudy environment, this study proposes the Electric Earthworm Optimization technique (EEOA), a novel multi-objective job scheduling approach. EEOA, a hybrid of the Earthworm Optimization Algorithm (EOA), and the Electric Fish Optimization Algorithm (EFO), enhances EFO's exploitation capabilities. Kumar and Karri (2023) implement EEOA, which is an attractive approach to cloud-fog job scheduling that is both efficient and economical. Significant gains in contrast to current techniques are shown in simulations utilizing real-world workloads like CEA-CURIE and HPC2N. Gains in efficiency of up to 89%, reductions in energy usage of 94%, and savings of 87% in overall cost are all part of these enhancements.

The explosive growth of IoT data creates a burden on the cloud, hindering QoS. To address this, fog computing extends computing capabilities to the network edge. Scheduling tasks in fog environments faces challenges due to heterogeneous fog nodes and dynamic user demands. This article presents a comprehensive literature review, classifying task scheduling algorithms by approach, highlighting frequently used parameters, comparing available simulation tools, and identifying open issues and challenges to guide future research efforts in fog computing task scheduling is written by Saad et al. (2023).

Due to the explosive growth of IoT and its high data demand, cloud computing struggles to support real-time applications with low latency. Fog computing emerges as a solution, enabling data processing near edge devices for faster response times. Scheduling tasks effectively on fog nodes with limited resources is crucial. This article proposes EETSPSO, an energy-efficient task scheduling algorithm based on Particle Swarm Optimization. EETSPSO outperforms existing algorithms like BLA and MPSO by minimizing makespan (6.39%−4.71%), reducing energy consumption (9.12%−11.47%), and decreasing execution time (9.83%−6.32%) and this is work is carried by Vispute and Vashisht (2023).

Fog computing helps improve the quality of service (QoS) for IoT applications by enabling task offloading near data sources. However, reducing delay remains challenging due to resource limitations and workload imbalance. To address these issues, this article proposes a dynamic collaborative task offloading (DCTO) approach that dynamically derives the offloading policy based on fog device resources is presented by Tran-Dang and Kim (2023). Tasks can be executed by one or multiple fog devices through parallel subtask execution to reduce delay. Extensive simulations show that DCTO significantly reduces average delay in systems with high request rates and heterogeneous fog environments compared to existing solutions. Additionally, DCTO's low computational complexity allows for online implementation. Extending cloud computing, fog computing offers lower latency and resource utilization improvements for IoT devices. Task scheduling in fog maps tasks to appropriate fog nodes to optimize resource usage and reduce IoT device costs. This article focuses on scheduling offloaded tasks for multiple users.

Formulating the problem as a combinatorial optimization, the article proposes an improved integer particle swarm optimization method for efficient solution. Compared to the traversal method, the He and Bai (2023) proposed algorithm achieves 90% faster runtime while still finding an approximate optimal solution, demonstrating its effectiveness for fog computing task scheduling optimization.

This article addresses the challenge of dynamic workflow scheduling in fog computing, where existing methods only consider cloud servers and ignore mobile and edge devices. A new problem model and simulator are presented that consider all three types of devices as a single system for task execution. A novel Multi-Tree Genetic Programming (MTGP) method is proposed by Xu et al. (2023) to automatically evolve scheduling heuristics for real-time decision-making at routing and sequencing points. Experiments show that MTGP significantly outperforms existing methods, achieving up to 50% reduction in makespan across different scenarios.

The increasing number of IoT devices using cloud services has led to latency issues. Fog computing, which places processing resources closer to users at the network edge, emerges as a solution to reduce latency and improve user experience. However, minimizing latency without increasing energy consumption requires a powerful scheduling solution. In order to strike a compromise between energy usage and response time, Khiat et al. (2024) introduce GAMMR, a new genetic-based method for job scheduling in fog-cloud situations. Simulations on different datasets show that GAMMR achieves an average 3.4% improvement over the traditional genetic method.

Workflow scheduling in cloud-fog settings is the difficult subject of this study. Bansal and Aggarwal (2023) designed a novel hybrid approach called the Particle Whale Optimization Algorithm (PWOA) to overcome the drawbacks of earlier algorithms like PSO and WOA. Combining the best features of PSO and WOA, PWOA enhances exploration and exploitation capabilities. Simulation results demonstrate that, across a range of scientific processes, PWOA minimizes both the total execution time and cost more effectively than PSO and WOA. It might be a good substitute for efficient workflow scheduling because of this. For application in fog-cloud computing settings, this article introduces a novel scheduling method called PGA. When optimizing task execution, the PGA algorithm considers not only energy consumption and deadline compliance but also the processing capability of the cloud and the resource limits of fog nodes. To achieve this goal, it uses a genetic algorithm in conjunction with job prioritization to distribute tasks to the right nodes in the cloud or fog. Hoseiny et al. (2021) conduct thorough simulations to show that PGA outperforms current techniques. Several researchers have used varying numbers of goals in an effort to solve the multi-objective optimization issue in workflow applications. For efficient resource allocation in workflows, this research suggests a hybrid meta-heuristic GA-PSO method. The suggested strategy leverages the characteristics of diverse jobs and nodes to achieve three objectives: reduce execution time, minimize reaction time, and lower overall completion time.

The article by Walia et al. (2023) provides a thorough examination of the most advanced solutions available, including both AI and non-AI approaches. It includes detailed discussions on related quality of service measures, datasets, constraints, and issues. Each categorized resource management problem is accompanied with mathematical formulas, which introduce a quantitative aspect to the examination. The study concludes by highlighting potential areas for future research, advocating for the incorporation of advanced technologies such as Serverless computing, 5G, Industrial IoT (IIoT), blockchain, digital twins, quantum computing, and Software-Defined Networking (SDN) into existing Fog/Edge paradigms. This integration aims to improve business intelligence and analytics in IoT-based applications. In summary, the article provides a thorough examination, analysis, and plan for tackling obstacles in managing Fog/Edge resources for IoT applications.

Kumar et al. (2023a) discuss the shortcomings of the cloud model in fulfilling the latency requirements of Industrial IoT (IIoT) applications. This article presents a new AI-based framework designed to optimize a multi-layered integrated cloud-fog environment. The system especially focuses on making real-time choices on offloading tasks. The framework integrates a fuzzy-based offloading controller and employs an AI-based Whale Optimization Algorithm (WOA) to increase decision-making for enhanced Quality-of-Service (QoS) parameters. The experimental findings reveal substantial improvements, such as a 37.17% drop in the time taken to complete a task, a 27.32% reduction in the amount of energy used, and a 13.36% decrease in the cost of executing the process, when compared to the standard reference methods. In summary, the proposed framework demonstrates the capacity of AI-driven solutions to enhance resource management in IIoT applications inside a fog computing environment.

Kumar et al. (2021) provide ARPS, a system that enables autonomous allocation and organization of resources on cloud platforms. ARPS is a system that aims to effectively allocate cloud services to fulfill the Quality of Service (QoS) needs of different end-users. It focuses on optimizing both execution time and cost at the same time. By using the Spider Monkey Optimization (SMO) method, this system tries to address a multi-objective optimization issue. Through rigorous simulation study using Cloudsim, it has been shown to outperform four other current mechanisms. On summary, ARPS offers a proficient approach to enhance resource allocation and scheduling on cloud platforms.

Kumar et al. (2023b) presents a system for predicting workload and allocating resources in fog-enabled Industrial Internet of Things (IoT). By using a sophisticated autoencoder model, the system predicts workloads and adjusts the number of fog nodes (FNs) accordingly. Additionally, it incorporates the crow search algorithm (CSA) to find the most effective FN. By conducting simulation study, the suggested scheme demonstrates superior performance compared to current models in terms of execution cost, request rejection ratio, throughput, and response time. This architecture provides a very effective approach for strategically positioning FNs to execute dynamic industrial IoT workloads with maximum efficiency.

The study focuses on the pressing issue of resource scheduling in cloud computing, which arises from the increasing need for on-demand services and the diverse characteristics of cloud resources (Kumar et al., 2019). Cloud services encounter inefficiencies when scheduling mechanisms are unable to effectively balance resource utilization, resulting in either a decline in service quality or resource waste. The main objective of scheduling algorithms is to evenly divide various and intricate jobs across cloud resources, while minimizing imbalance and optimizing crucial performance characteristics like as reaction time, makespan time, dependability, availability, energy consumption, cost, and resource utilization. The literature review categorizes current scheduling algorithms, including heuristic, meta-heuristic, and hybrid methods, emphasizing their strengths and weaknesses. The work seeks to function as a methodical and all-encompassing reference for novice researchers in the cloud computing domain, promoting more advancement in scheduling methodologies.

This article tackles the complex scheduling of real-time tasks on multiprocessors using a new algorithm called mohGA (Yoo and Gen, 2007). Combining GA and SA's strengths, mohGA efficiently finds near-optimal schedules while optimizing conflicting objectives like tardiness and completion time. Simulation results show mohGA outperforms existing methods, making it a promising approach for real-time task scheduling in various applications.

“Network Models and Optimization: Multiobjective Genetic Algorithm Approach” provides a thorough and up-to-date examination of the use of multi-objective genetic algorithms in solving diverse network optimization issues in numerous fields (Gen et al., 2008). The extensive scope of algorithms and applications covered in this resource, which includes basic principles such as shortest route issues and complex situations like airline fleet assignment, is suitable for both individuals seeking knowledge and professionals in the field. This book is a wonderful resource for anyone who want to comprehend and apply sophisticated network optimization approaches employing multi-objective genetic algorithms.

The author in his book (Gen and Yoo, 2008) examines the use of Genetic Algorithms (GA) in scheduling soft real-time jobs on multiprocessors. The objective is to minimize tardiness while reducing the complexity associated with conventional approaches. The benefit of GA stems from its multifaceted approach that integrates principles and heuristics, providing more intricate solutions for this NP-hard issue. The chapter offers a complete method to effective job scheduling with decreased complexity by addressing continuous, homogeneous, and heterogeneous real-time systems.

The outcomes produced by algorithms based on the GA algorithm are, in essence, preferable to those produced by other algorithms when the total number of process repetitions exceeds a specifically defined threshold. Conversely, as the number of iterations of the process increases, the GA-based meta-heuristic algorithm will require an extended duration to generate the optimal solution. In addition, PSO-based meta-heuristic algorithms exhibit superior performance in a reduced time period in comparison to alternative approaches. Due to the rapid convergence of PSO-based algorithms toward a solution, however, the results' dependability may be compromised. This rapid convergence could cause the algorithms to become stuck in the solution that is locally optimal.

As a result, the suggested algorithm differentiates itself by using the qualities that are associated with both the GA and the PSO algorithms. When compared to previous algorithms with the same goals, it is anticipated that the Hybrid GA-PSO method would function much quicker with a wider variety of workflow application sizes. In addition, the Hybrid GA-PSO method does not necessarily become stuck in the locally optimum solution since it makes use of the hybrid approach as it combine the GA with other optimization algorithms, such as local search methods (PSO), to leverage their respective strengths, which increases the precision of the answers provided and stops the algorithm from becoming trapped in the solution that is optimum for the local environment.

## 3 Problem statement

Current scheduling methods often fail to adequately handle important factors in fog computing, such as response time, energy cost, and resource utilization. We need a more advanced and adaptable task scheduling solution since fog settings are dynamic, with different workloads and different hardware capabilities.

The two main ideas in genetic algorithms (GAs) that stand for the harmony between finding new solutions and improving old ones are exploration and exploitation (Gen and Lin, 2023a). In order to find superior answers in other areas, exploration must extensively explore the search space. Taking use of what is already known about potential solutions allows you to boost their efficiency. An algorithm that puts too much emphasis on exploration runs the risk of squandering time and energy looking in fruitless corners of the search space. On the other side, if the algorithm starts focusing on exploitation too soon, it might end up convergent to a local optimum before it ever starts looking for a better solution.

In extreme cases of natural selection, it is advantageous to reproduce individuals with high fitness levels. This means other, perhaps more productive areas of the search space can be overlooked when the space rapidly converges on a local limit. The current state of the population should inform how the selection pressure is adjusted. Here we may find a happy medium between exploiting and exploring. There are a few drawbacks to be mindful of, such premature convergence, but the genetic algorithm (GA) has proved effective in solving many optimization problems. The GA stops looking for potential productive regions of the search space because it becomes stuck at a local optimum. Genetic algorithms' (GAs) primary drawback is the possibility of over-convergence, which prevents them from exploring the search space for optimal solutions and instead keeps them stuck in a local optimum. The GA's effectiveness is diminished, leading to frequent poor results.

It is possible to avoid early convergence in GA by using hybrid techniques. To get the most out of GA, try combining it with other optimization techniques, including local search approaches. Both the GA and local search algorithms can work together to improve GA-found solutions; the GA can even provide new genetic material to sidestep local optima. The benefits of GA-PSO, a combination of genetic and particle swarm optimization, outweigh those of employing either approach alone. An improved search process and maybe superior solutions are the results of the hybrid strategy, which merges GA's exploration skills with PSO's exploitation characteristics (Shami et al., 2023). In order to effectively explore the search space, GA makes use of its mutation and crossover operators. Based to its information-sharing mechanism and velocity updates, PSO efficiently uses promising portions of the search space to quickly converge toward locally optimum solutions.

## 4 The proposed algorithm

### 4.1 Key components of the proposed algorithm

**Chromosome representation**: incorporate critical data like task priority, execution timings, fog node assignments, and other relevant information into an efficient chromosomal representation that encodes task scheduling solutions.

**Genetic algorithm (GA) components**: utilize genetic operators such as mutation and crossover to provide a diverse set of solutions for job assignment. Build a comprehensive fitness function that considers all relevant metrics, including resource utilization, energy usage, response time, and more.

**Particle swarm optimization (PSO) components**: use particles to represent potential solutions to the assignment's challenge. Using location and velocity updates, you may guide particles toward the optimal solutions in the solution space.

**Hybridization strategy**: by combining the strengths of GA and PSO, you can speed up the exploration process and get closer to the best possible answers.

**Dynamic adaptation**: it may be difficult for the traditional task scheduling approaches to adapt the dynamic and diverse distributed systems in the fog computing settings. Create dynamic methods that the algorithm may use to adapt to changes in the fog environment, such changes in workload, network condition, and resource availability.

**Expected outcomes**: the proposed Blended GA-PSO algorithm anticipates providing a highly adaptive and efficient task scheduling solution for fog computing environments. The outcomes are expected to include improved system performance, reduced response times, and enhanced resource utilization.

**Figure 2:**
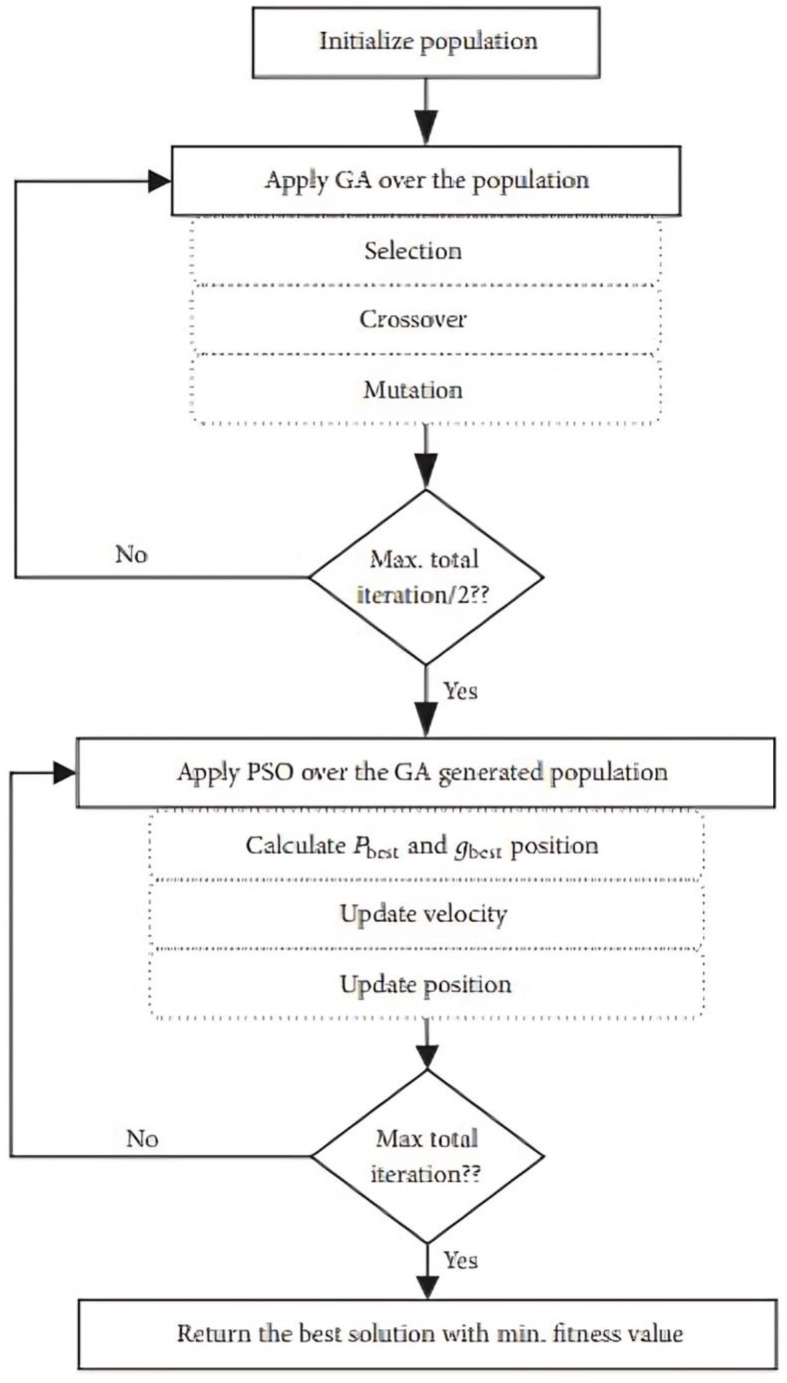
Workflow of GA-PSO Algorithm.

Figure 2 illustrates the primary operations involved in the GAPSO algorithm. The GA-PSO method begins by producing a random population (Mortaheb and Jankowski, 2023), and one of the parameters of the algorithm is a certain number of iterations that must be completed before the process may proceed. The population demonstrates that there is more than one approach to solving the issue of workflow tasks, and each approach is a method for allocating all of the workflow jobs to the VMs that are now accessible. The GA algorithm is run on the initialized population for the initial number of iterations. The total iterations are set to (n), then the GA algorithm and PSO algorithm will be executed half times of n. Iteration of the form (n/2) was chosen since it makes the suggested algorithm to improved performance compared to using either GA or PSO alone. GA excels at exploration, while PSO shines at exploitation. By allocating equal time, the hybrid algorithm leverages both capabilities effectively simpler to grasp, which is why it was used.

Experiments demonstrated that the GA-PSO algorithm functioned most effectively when the number of iterations was divided between the GA algorithm and the PSO algorithm in an equal manner. Additionally, it is well knowledge that the GA and the PSO need a great deal of function evaluations.

This is due to the fact that each has to consider the objective of each individual participant in the population represented by the current illustration. As a result, reducing the population size in a genetic algorithm or particle swarm optimization is a popular technique for keeping the GA or PSO's performance from deteriorating in terms of how accurate the findings are and how quickly the rate of reduction is.

Pareto optimality is a state in which a group of solutions exists where no individual solution can be enhanced without causing a deterioration in another solution (Gen and Cheng, 2000; Zhang et al., 2022; Gen and Lin, 2023b). Task scheduling involves the identification of schedules that optimize many goals concurrently, such as minimizing makespan and resource utilization. The proposed Hybrid GAPSO algorithm can be customized to identify Pareto optimum solutions using the following methods: Creating several fitness functions that reflect distinct aims, Employing multi-objective selection procedures that prioritize solutions that provide compromises among goals.

Within the framework of the genetic algorithm (GA), also known as the genetic algorithm, the solutions are referred to as chromosomes. These chromosomes are enhanced with each iteration of the algorithm by making use of GA operators including selection, crossover, and mutation (Gen and Lin, 2023a). These GA operators are all part of the genetic algorithm. The generated chromosomes are what is sent into the PSO algorithm after the first half of the set number of iterations has been finished. In the PSO method, chromosomes are referred to as particles, and with each iteration of the PSO algorithm (Shami et al., 2023), these particles go through a process that results in a little but noticeable improvement. In order to accurately reflect the answer to the workflow task issue, the particle that has the lowest fitness value has been chosen.

### 4.2 Initializing population

The search for a solution at the beginning of the iteration is carried out in a random fashion. Upon completion of the first cycle, a number of new populations are generated, and then those populations are recursively improved by making use of the previously found solutions to provide a collection of proposed solutions.

Chromosome is the term used to refer to the population in the GA method. The number of workflow jobs is proportional to the length of the chromosomes, and the genes that make up each chromosome are meant to stand in for the various virtual machines. The chromosomes that were created at random serve as the input for the GA-PSO method that was just developed. These solutions will be chosen based on the performance of the GA algorithm. Algorithm 1 provides a visual representation of the whole initialization population phase.

**Algorithm 1:** The initializing population.

Algorithm: InitializePopulationAndParticles
Input: Population size (popSize), Number of tasks
(numTasks), Number of fog nodes (numFogNodes)
1. InitializePopulation(popSize, numTasks,
numFogNodes):
For each individual in the population:
Generate a chromosome with random task
assignments to fog nodes.
2. InitializeParticles(popSize, numTasks,
numFogNodes):
For each individual in the population:
Create a particle with a position
equal to
the chromosome and a random velocity.

### 4.3 Utilizing the GA algorithm

Applying the GA to the whole population that has been created for (n/2) of the specified iterations is the first stage in the procedure. In order to solve the scheduling issue, it is required to develop the best answer possible from all of the alternatives. For the next (n/2) rounds of the specified procedure, the PSO is applied to the whole population that the GA algorithm generates. The PSO approach, which retains both the best and worst solutions in memory, may be useful for attaining quick convergence on the optimum solution when the GA algorithm yields inadequate outcomes.

The total number of workflow jobs is equivalent to the number of chromosomes that are utilized as symbols for the answers provided by the genetic algorithm (GA), which define the scheduling solution for our issue. Each chromosome has a number of genes that stand in for the hosts' virtual machines (VMs). During each cycle of the GA, the chromosomes are passed via three distinct operators: the crossover, mutation, and selection operators. The selection operator is the GA algorithm's initial operator. In order for the algorithm to generate the succeeding generation of chromosomes, this operator is in responsible of picking various options from the pool of already-existing chromosomes in order to solve the problem.

### 4.4 Selection operator

In the genetic algorithm known as the GA, each iteration does not include the evolution of all of the produced chromosomes via the GA operators. As a result, the chromosomes go through a process known as the tournament selection in order to choose the most advantageous chromosome from among a set of chromosomes. After the completion of a number of competitions involving a small number of chromosomes, the function chooses an id at random. The chosen ids each stand for an index that refers to one particular chromosome out of a larger collection of chromosomes. According to Algorithm 2, the best chromosome in the group is chosen to be the crossover operator. This decision is based on the chromosome's overall fitness.

**Algorithm 2:** The selection operator (tournament selection).

Algorithm: TournamentSelection
Input: Population, Tournament size (tournamentSize)
1. DividePopulationIntoGroups(Population,
tournamentSize):
//Divide the population into groups of
equal size
(tournament size)
For each group in the population:
GroupMembers =
RandomSubset(Population,
tournamentSize)
2. For each group in the
divided population:
//Choose the individual with the best
fitness as the winner
of the tournament
Winner = SelectWinner(GroupMembers)
3. Return two tournament winners
for crossover:
TournamentWinners =
RandomSubset(Winners, 2)
Return TournamentWinners

### 4.5 Fitness function

The primary objective of task scheduling in a fog computing environment is to minimize the amount of time required to complete a given activity, respond to a task, and finish a task. Algorithm 3 provides a visual representation of the whole fitness function.

**Algorithm 3:** The fitness function.

Algorithm: CalculateFitness
Input: Chromosome, Fog Node Capabilities, Task Assignments
1. For each chromosome in the population:
//Calculate execution time, response
time, and
completion time based on task
assignments and fog
node capabilities
CalculateExecutionTime(chromosome,
f ogNodeCapabilities, taskAssignments)
CalculateResponseTime(chromosome,
fogNodeCapabilities, taskAssignments)
CalculateCompletionTime(chromosome,
fogNodeCapabilities, taskAssignments)
//Calculate fitness as the inverse of
the sum of
execution time, response time, and
completion time
Fitness =
CalculateFitnessValue(executionTime,
responseTime, completionTime)
2. Return fitness value.
Return Fitness

The task execution time, shows how long it takes task i to run on virtual machine j, based on the following Equation 1.


(1) $$\begin{array}{l} E_{ct}\left(i,j\right)=\frac{t_{length\left(i,j\right)}}{vm_{comp\left(i,j\right)}} \end{array}$$

Where *t*_*length*(*i,j*)_ is the duration of the job necessary to execute the instruction, vm represents the computational capacity of the *vm*_*comp*(*i,j*)_ virtual machine based on the following Equation 2.


(2) $$\begin{array}{l} vm_{comp\left(i,j\right)}=vm_{pesNumber\left(i,j\right)}^{*}vm_{mips\left(i,j\right)} \end{array}$$

Where *vm*_*pesNumber*(*i,j*)_ is the number of CPUs in virtual machine j, *vm*_*mips*(*i,j*)_ represents the processing power of virtual machine j.


(3) $$\begin{array}{l} RT\left(i,j\right)=FT\left(i,j\right)-ST\left(i,j\right) \end{array}$$

Where *RT*(*i, j*) is the response time of all jobs i executing on resources j. *FT*(*i, j*) is the task's completion time and *ST*(*i, j*) is its beginning time as shown in Equation 3.

The total amount of time it takes to finish a job *FT*(*i, j*) is the addition of the amount of time it takes to transfer the task *RT*(*i, j*) and the amount of time it takes to run the task on the virtual machine *E*_*ct*_(*i,j*) as shown in Equation 4.


(4) $$\begin{array}{l} FT\left(i,j\right)=RT\left(i,j\right)+E_{ct}\left(i,j\right) \end{array}$$

### 4.6 The crossover operator

The crossover operator seeks to build new chromosomes by rearranging the genes on every set of chromosomes in a different order. A number denoting the stage at which each chromosome is split in half is chosen at random from the range of the total number of genes on each chromosome in the crossover approach. A child chromosome with two sections containing the genes, or VMs, of both parents' chromosomes is the result of the crossover. The first group of virtual machines (VMs) use the first chromosome up to the random number-determined index. The second chromosome is home to the second set of virtual machines, which start at the randomly selected index and go all the way to the end of the chromosome. An example of how the crossover strategy could be used is shown in Algorithm 4.

**Algorithm 4:** Cross over operator.

Algorithm: Crossover
Input: Parent1, Parent2
1. SelectTwoChromosomesForCrossover:
//Randomly select two chromosomes
for crossover
Parent1 = SelectRandomChromosome()
Parent2 = SelectRandomChromosome()
2. ChooseRandomCrossoverPoint:
//Choose a random crossover point
within the
chromosome length
CrossoverPoint =
RandomInteger(1, ChromosomeLength)
3. CreateOffspringChromosomes:
//Create two offspring chromosomes by
swapping genes
after the crossover point between
the parents
Offspring1 =
Concatenate(Parent1[1:CrossoverPoint],
Parent2[CrossoverPoint+1:])
Offspring2 =
Concatenate(Parent2[1:CrossoverPoint],
Parent1[CrossoverPoint+1:])
4. ReturnTheTwoOffspringChromosomes:
//Return the two offspring chromosomes
Return Offspring1, Offspring2

### 4.7 The mutation operator

The mutation operator's goal is to induce unexpected mutations into the new chromosomes created by the preceding crossover operator. These newly formed chromosomes ought to be more fit than the ones that are currently there. The mutation rate variable decides whether or not a mutation will really occur. The mutation operator operates on the chromosome that was returned by the selection procedure. The process of mutation begins with a number that is created at random in such a way that it is either lower than or the same as the mutation rate. It is necessary to determine whether or whether two genes, known as VMs, located on the same chromosome are distinct from one another. If they are identical, their places will be altered in order to build a new chromosome, indicating a different work allocation across the available virtual machines (VMs). The chromosome that has been created is then sent on to the subsequent step of the process. Algorithm 5 provides an illustration of the method's implementation, which is known as mutation.

**Algorithm 5:** Mutation operator.

Algorithm: Mutation
Input: Chromosome, Mutation Rate
1. For each gene in the chromosome:
//With a small probability (mutation
rate), randomly
choose a new fog node for the task
For each gene in the chromosome:
If RandomNumber(0, 1) < MutationRate:
MutateGene(chromosome, gene)
2. Return the mutated chromosome.
Return Chromosome

### 4.8 Implementing the PSO algorithm

The solutions obtained by the GA algorithm are then put into the PSO algorithm, together with the remaining iterations indicated, to select the optimal solution from among the solutions generated by the GA technique. This is done to identify which of the solutions produced by the GA technique is the best solution. The solutions are referred to as particles in the PSO approach, the persons who make up each particle stand in for the VMs, and the index of each VM stands in for a workflow job. The PSO algorithm is divided into many sections, all of which will be explained in this article.

### 4.9 Evolve (gbest) and (pbest) of the particles

Each iteration results in the production of a new generation of the particles by basing that generation's velocity and location on the results of the previous iteration. The values of (gbest) and (pbest) are created during each iteration; the changes in particle velocity and placement are reliant on these values. Algorithm 6 shows how to implement obtaining the best gbest and best pbest values of the particles. The values of (gbest) and (pbest), which are dynamically modified over the algorithm's iterations, determine the forward movement of the particles in the PSO algorithm. These values are always shifting. The solutions that are generated by the GA method are identical to the values of pbest[k] for the very first iteration, where k is the variable that is utilized to distinguish one solution from another. This holds true for all of the iterations that follow. The alternative that results in the highest possible score (gbest) is the one that has the lowest possible fitness value.

**Algorithm 6:** Evolve (gbest) and (pbest) of the particles.

Algorithm: ParticleSwarmOptimization
Input: Population, PSO Parameters
1. IdentifyGlobalBest(Population):
/Identify the best individual in the
current population
(global best)
GlobalBest =
FindBestIndividual(Population)
2. For each particle in the swarm:
//Identify the best individual it has
encountered so far
(personal best)
PersonalBest =
FindBestIndividual(ParticleHistory)
//Update velocity and position based on
personal best,
global best, and other PSO parameters
UpdateVelocityAndPosition(Particle,
PersonalBest,
GlobalBest, PSOParameters)

In addition, throughout each iteration, a comparison is made between the particles that were previously formed and the particles that are now being generated, and this comparison is based on the fitness value. The location that has the particle with the highest value of fitness is (pbest). The (gbest) saves the most suitable particle from the entire production of particles in every repetition by contrasting their fitness value to the value of the particle with the best value in the previous iteration (Pbest). The comparison procedure guarantees that all of the particles are progressing toward the solution that is considered to be the best in order to arrive at the solution that is considered to be the optimum one.

The pbest and gbest are crucial in guiding the particles toward promising regions of the solution space. The pbest allows each particle to remember its own historical best position, while the gbest guides the entire swarm toward the overall best position discovered by any particle. The process of updating pbest and gbest is straightforward:

#### 4.9.1 Update individual best (pbesti)

If the fitness (or objective value) of the current position xi(t) is better than the fitness of pbesti, update pbesti to xi(t) as shown in Equation 5.


(5) $$pbesti=\{\begin{array}{c} xi\left(t\right)\;if\;fitness\left(xi\left(t\right)\right)<fitness(pbesti)\\ pbesti\;otherwise\; \end{array}$$

#### 4.9.2 Update global best (gbest):

If the fitness of pbesti is better than the fitness of gbest, update gbest to pbesti as shown in Equation 6.


(6) $$pbesti=\{\begin{array}{c} pbesti\;if\;fitness\left(pbesti\right)<fitness(gbest)\\ gbest\;otherwise\; \end{array}$$

These updates ensure that the particles converge toward optimal solutions in the search space over iterations, leveraging both personal and swarm-wide historical information.

#### 4.9.3 Update the velocity and position matrix

In particle swarm optimization (PSO), the evolution of particles is governed by updating their positions and velocities based on the best historical positions, both individual (pbest) and global (gbest). Let's denote xi as the position vector of particle i, vi as the velocity vector of particle i, pbesti as the best historical position of particle i, and gbest as the best position among all particles in the entire swarm.

The equations for updating the velocity and position of each particle in a basic form of PSO are as follows:

#### 4.9.4 Velocity update (**vi**)


(7) $$\begin{array}{ll} vi\left(t+1\right)= & w\cdot vi\left(t\right)+c1\cdot rand1\cdot \left(pbesti-xi\left(t\right)\right)+c2\cdot rand2\cdot \\ \left(gbest-xi\left(t\right)\right) \end{array}$$

^*^ w is the inertia weight, controlling the impact of the previous velocity.

^*^ c1 and c2 are the acceleration coefficients for personal and global influence, respectively.

^*^ rand1 and rand2 are random values between 0 and 1.

#### 4.9.5 Position update (**xi**)


(8) $$\begin{array}{l} xi\left(t+1\right)=xi\left(t\right)+vi\left(t+1\right) \end{array}$$

These Equations 7, 8 reflect the collaborative exploration of the solution space by particles. The first term in the velocity update equation represents the inertia of the particle, the second term is the attraction to the particle's own best position (exploitation), and the third term is the attraction to the swarm's best position (exploration).

The velocity of each particle is altered proportionally throughout each iteration after the random creation of the beginning values for the velocity and position of the particles, as well as the computation of both (pbest) and (gbest). An example of how the update procedure for the velocity matrix should be implemented is shown in Algorithm 7, which can be found here.

**Algorithm 7:** Update velocity and position matrix.

Algorithm: ParticleSwarmOptimization
Input: Swarm, Inertia Weight, Cognitive Acceleration Coefficient, Social Acceleration Coefficient, Velocity Bounds
For each particle in the swarm:
//Update velocity based on
current velocity,
inertia weight, cognitive and
social acceleration
coefficients, distances to personal and
global best
positions
UpdateVelocity(Particle, InertiaWeight,
CognitiveAccelerationCoefficient,
SocialAccelerationCoefficient)
//Bound velocity within a predefined range
BoundVelocity(Particle, VelocityBounds)
//Update position based on the
updated velocity
UpdatePosition(Particle)
//If a position violates fog node resource
constraints, repair it by re-assigning
tasks while
minimizing fitness degradation
RepairPosition(Particle)

The method, which includes changing the speed of the particles, tries to produce another type of particle from the different portions of the VMs that is more suited than the previous one. This new generation will be produced as a result of the procedure that involves updating the velocity of the particles.

A comparison is made between the value of each individual included inside the particles and its value from the prior iteration of the procedure, which was indicated by the notation pbest. When both individuals in (pbest) and the particle have the same velocity, the velocity value for each person is reduced; otherwise, the velocity amount is increased. In a similar manner, a comparison is being made between each individual inside the particles and the values that they had during the last iteration of the process (gbest). When both persons in (gbest) and the particle have the same velocity, the value of the individual's velocity is lowered; otherwise, the value of the individual's velocity is raised. Therefore, the location of the virtual machines (VMs) that make up each particle is altered in accordance with the modified values of the velocity.

Two virtual machines (VMs) with maximum velocity values are switched within each particle in the newly created population. The word “maximum” serves as a symbol for the GAPSO algorithm's termination criteria, which is the condition that must be met before the method can be considered complete. When all of the termination conditions have been satisfied, the scheduling solution for the workflow application will be changed to the one that was devised during the most recent iteration of the process and will have the lowest fittness value among the population. If this is not the case, the values of (gbest) and (pbest) continue to iteratively change until the termination condition is met.

Repeat all steps until a termination criterion is met (e.g., maximum iterations). Return the best chromosome in the population as the optimal task allocation solution.

Algorithm 8 provides a visual representation of the whole GA-PSO method.

**Algorithm 8:** The proposed algorithm.

**Input**:
- Workflow W {N, E}
- Set of resources {VM1, VM2, ..., VM}
- p: Population size
- n: Number of iterations
**Output**:
- gbest: The best solution to allocate W over VM
**Algorithm**:
1. Initialize population by randomizing p solutions.
2. For i = 0 to p:
2.1 Randomly initialize the population.
2.2 End loop.
3. Initialize a counter n_half = 0.
4. While n_half is less than n/2:
4.1 For each Chromosomej and Chromosomei in
population:
4.1.1 Apply tournament selection
to Chromosomej.
4.1.2 Apply tournament selection to
Chromosomei.
4.1.3 Apply crossover to generate
offspring_chromosomej.
4.1.4 Apply mutation to generate
Newchromosomej.
4.2 End inner loop.
5. While not Reach n:
5.1 Initialize particles' position and
velocity randomly.
5.2 Calculate the gbest and Pbest values
for each
particle.
5.3 While not Reach n:
5.1.1 Update particle velocity matrix using
particlej's velocity update.
5.1.2 Update particle position matrix using
particlej's position update.
5.1.3 Repeat inner loop.
5.4 End outer loop.
6. Output the best solution gbest.
7. End algorithm.

The above proposed algorithm can be mathematically expressed as shown below:

**Sets:**

W = (N, E) : Workflow represented as a directed graph with node set N and edge set E

VM = {VM1, VM2, ..., VMm} : Set of m virtual machines

**Parameters:**

p ε N : Population size, a positive integer

n ε N : Number of iterations, a positive integer

**Variables:**

X_i: Solution (chromosome) i, represented as a vector of task-to-VM assignments

V_i: Velocity of particle i in PSO

gbest: Global best solution

Pbest_i: Personal best solution of particle i

**Initialization:**

X_i ← random_initialization(W, VM) for i = 1 to p

n_half ← 0

**Genetic Algorithm Phase:**

While n_half < n/2:

For each pair of chromosomes X_j, X_i ← population:

P1, P2 ← tournament_selection(population)

offspring_X_j ← crossover(P1, P2)

new_X_j ← mutation(offspring_X_j)

n_half ← n_half + 1

**Particle Swarm Optimization Phase:**

X_i ← random_initialization(W, VM), V_i ← random_vector() for i = 1 to p

Calculate gbest, Pbest_i for each particle

While n_half < n:

For each particle j:

V_j ← update_velocity(V_j, Pbest_j, gbest)

X_j ← update_position(X_j, V_j)

Recalculate gbest, Pbest_i if necessary

**Output:**

Return gbest

**Encoding Algorithm:**

The encoding scheme defines how tasks and resources are represented in the chromosomes (solutions) used by the hybrid GA-PSO algorithm. Here's an approach:

- Chromosome structure: Each chromosome is an array of N genes, where N is the number of tasks in the workflow.
- Gene representation: Each gene represents the assigned resource for a specific task. This can be achieved via direct encoding in which the gene value directly corresponds to the resource ID (VM1, VM2, etc.).

**Decoding Algorithm:**

The decoding algorithm interprets the encoded chromosomes back into actual task allocations on fog nodes. Here's the process:

- Iterate over each chromosome.
- For each gene i in the chromosome:

- Based on encoding scheme:
  - Direct Encoding: Look up the resource ID from the gene value (i).
-Assign the task i to the identified resource.

Sample Example:

Workflow W: 4 tasks (T1, T2, T3, T4).

Fog Nodes: 3 nodes (VM1, VM2, VM3).

Chromosome (Direct Encoding): (Chandra and Verma, 2023; Fanelli et al., 2023; Mortaheb and Jankowski, 2023)

Decoding Process:

T1 is assigned to VM2 (gene value 2).

T2 is assigned to VM1 (gene value 1).

T3 is assigned to VM3 (gene value 3).

T4 is assigned to VM2 (gene value 2).

The effectiveness of the proposed GA-PSO method for task scheduling in fog computing is confirmed by extensive tests carried out utilizing the iFogSim simulator. The studies conducted a performance comparison between GA-PSO and well-known algorithms like as GA, PSO, and PWOA. This comparison was carried out across multiple situations, including diverse data inputs and fog node configurations. The primary findings validate the feasibility of GA-PSO in several aspects:

**Superior performance**: GA-PSO shown superior performance compared to all other algorithms in terms of minimizing execution time, reaction time, and completion time for jobs. This shows its efficacy in identifying the most effective work assignments inside the fog environment.

**Effectiveness with heterogeneity**: GA-PSO demonstrated exceptional performance even in situations where there was a wide range of job complexity and various capabilities of fog nodes. This illustrates its capacity to adapt to real-world circumstances when resources and workloads are not always homogeneous.

**Dynamic adaptation**: the algorithm has an inherent mechanism for dynamic adaptation, enabling it to modify its behavior in response to changing variables in the fog environment, including changes in workload, network availability, and resource availability. This functional characteristic guarantees consistent and efficient operation even under changing circumstances.

In summary, the experimental findings strongly support the notion that the GA-PSO algorithm, as described, is both theoretically robust and practically successful in real-world fog computing environments. The exceptional performance, versatility, and efficacy across many workloads establish its potential as a key tool for task scheduling in fog computing applications.

### 4.10 Performance evaluation and results

In order to test the proposed algorithm, the ifogsim (Gen and Lin, 2023a) simulator was used to implement the proposed algorithm. In order to determine how well the proposed hybrid GA-PSO algorithm performs, two experiments have been conducted and its obtained results have been compared with those of other task scheduling algorithms already in use, such as the GA proposed in Reddy et al. (2020), the PSO algorithm proposed in Jabour and Al-Libawy (2021) and PWOA algorithm (Bansal and Aggarwal, 2023). In order to assess the efficiency of the Hybrid GA-PSO algorithm that was proposed, this action was taken. The experimental parameters are clearly outlined in Table 1. For the first experiment, we came up with five scenarios in which the number of tasks was incremented by 100 in each scenario. The number of fog nodes remained constant at 55 throughout the entire experiment, and the obtained results are presented in **Table 4**. In the same way, we came up with five scenarios for experiment number two, with the number of fog nodes varying by 15 and the number of tasks remaining constant at 200 throughout the entire experiment. The outcomes are detailed in **Table 5**. These results are in depth discussed in results and discussion section.

**Table 1:** Experiment parameters.

**Experiment**	**Purpose**	**Data input parameters**	**Fog node parameters**
1	Heterogenous task	[100,100,500]	55
2	Heterogenous nodes	200	[15,15,75]

### 4.11 Simulation setup

In order to compare the proposed method to existing algorithms regarding the scheduling issue of workflows, we performed detailed tests on an efficient car parking real-world workflow application using the simulation settings (Gupta et al., 2017) listed in Tables 2, 3. The simulation parameters included the number of data inputs and the characteristics of fog nodes. In determining the attributes of the fog node and workflow application employed in the study, these factors were considered.

**Table 2:** Task characteristics.

**Parameters**	**Units**	**Type1 values**	**Type2 values**
Length	SI	[1,182, 4,090]	[5,232, 9,648]

**Table 3:** Fog node characteristics.

**Parameters**	**Units**	**Fog node values**
CPU length	MIPS	[500, 2,000]
Ram	MB	4,000
Uplink bandwidth	Mbps	10,000
Download bandwidth	Mbps	10,000

We conceived of a scenario in which parking spaces are captured on film by intelligent, high-definition cameras. The images are subsequently transmitted to the fog node. The fog node evaluates the state of the parking space by analyzing the images and presents visual representations of the parking space through a Wi-Fi-enabled smart LED that is affixed to the fog node (Gupta et al., 2017). Through the use of a proxy server, communication is established between the fog nodes and the cloud server. We established variables for parking spaces and the quantity of cameras within the simulation. As part of our experimental setup, five parking lots were established. Initially, between one hundred and five hundred cameras were installed in each parking lot for the purpose of capturing images of the parking area.

It is essential to note that a minimum of one fog node was generated for each distinct region. Subsequently, the number of fog nodes was expanded to facilitate the analysis of results obtained from various configurations. By virtue of their intelligent nature (equipped with WiFi capabilities) and microcontroller connectivity, we successfully implemented the cameras within the simulation environment and classified them as sensors in accordance with the guidelines outlined in Sheikh et al. (2023). We augmented the quantity of cameras in order to conduct an analysis of the data collected across diverse configurations and to assess the impacts on the fog node's execution time, response time, and completion time.

## 5 Results and discussion

### 5.1 Experiment one (number of tasks trade off)

**Figure 3:**
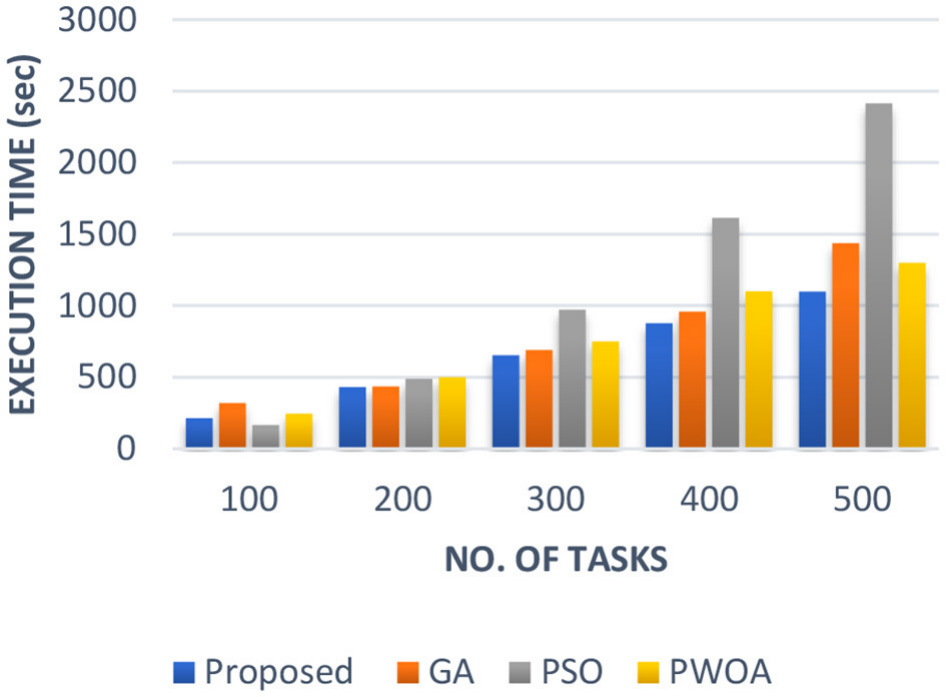
Comparison of Execution time (in second) of proposed Hybrid algorithm with conventional GA, Hybrid PWOA and PSO with different number of data input (cameras).

**Figure 4:**
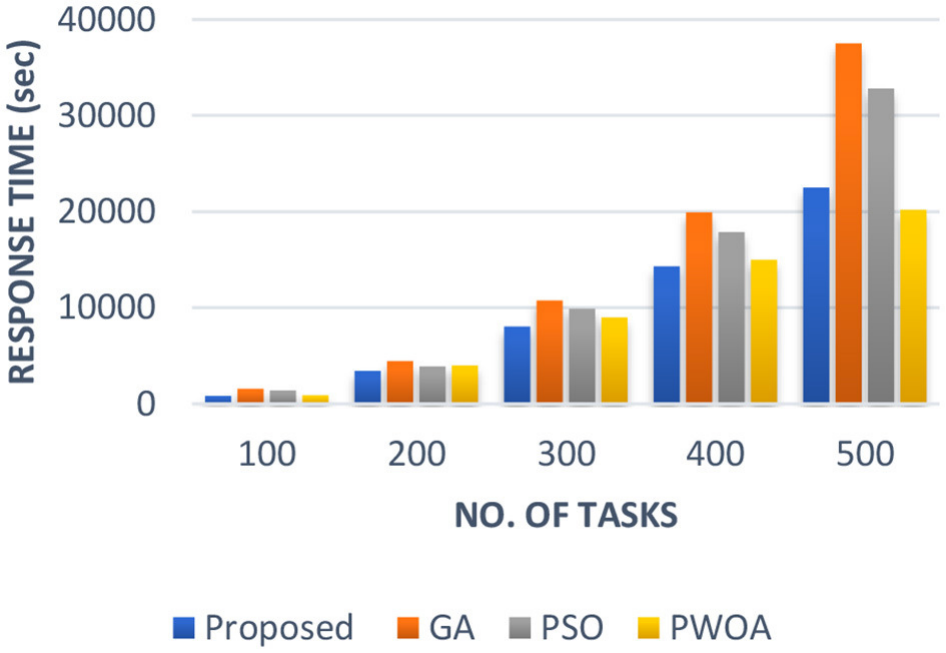
Comparison of Response time (in second) of proposed Hybrid algorithm with conventional GA, Hybrid PWOA and PSO with different number of data input (cameras).

**Figure 5:**
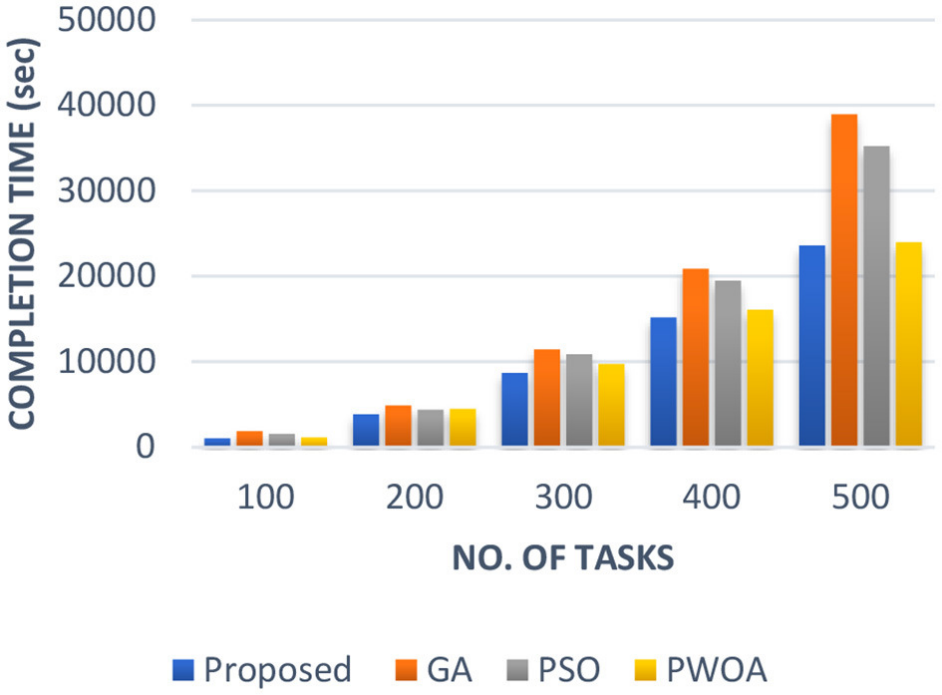
Comparison of Completion time (in second) of proposed Hybrid algorithm with conventional GA, Hybrid PWOA and PSO with different number of data input (cameras).

In Table 4 we have presented the outcomes of hybrid GA-PSO algorithm, GA algorithm, Hybrid PWOA algorithm, and PSO algorithm in terms of execution time, response time, and completion time when various data input are given. Figure 3 indicates the proposed hybrid algorithm results improved the execution time by 85.68% when compared with GA algorithm, by 84% when compared with Hybrid PWOA and by 51.03% when compared with PSO algorithm. Figure 4 indicates the hybrid GA-PSO algorithm results improved the response time by 67.28% when compared with GA algorithm, by 54.24% when compared with Hybrid PWOA and by 75.40% when compared with PSO algorithm. Figure 5 indicates the hybrid GA-PSO algorithm results improved the completion time by 68.69% when compared with GA algorithm, by 98.91% when compared with Hybrid PWOA and by 75.90% when compared with PSO algorithm.

**Table 4:** The result of experiment number one.

**Algorithm**	**No. of task**	**Execution time (sec)**	**Response time (sec)**	**Completion time (sec)**
**Scenario one**				
GA-P	100	213.58	827.37	1,040.95
GA		319.75	1,567.14	1,886.89
PSO		165.81	1,397.24	1,563.05
PWOA		245.30	900.50	1,148.75
**Scenario two**				
GA-P	200	430.92	3,432.93	3,863.84
GA		435.3	4,458.42	4,893.72
PSO		489.35	3,901.05	4,390.4
PWOA		500	4,000.2	4,500.2
**Scenario Three**				
GA-P	300	653.75	8,052.06	8,705.81
GA		690.52	10,759.93	11,450.45
PSO		972.37	9,912	10,884.37
PWOA		750.01	9,000.15	9,750.16
**Scenario Four**				
GA-P	400	877.62	14,320	15,197
GA		958.72	19,934.28	20,893.01
PSO		1,614.1	17,882.4	19,496.5
PWOA		1,100.62	15,000	16,100.62
**Scenario Five**				
GA-P	500	1,098.88	22,516.54	23,615.42
GA		1,437.36	37,530.06	38,967.42
PSO		2,414.29	32,822.27	35,236.56
PWOA		1,300	22,700.05	24,000.05

Therefore, GA-PSO is the most efficient algorithm in terms of execution time, response time and completion time, followed by PSO, Hybrid PWOA, and GA is the least efficient algorithm.

### 5.2 Experiment two (fog node trade off)

**Figure 6:**
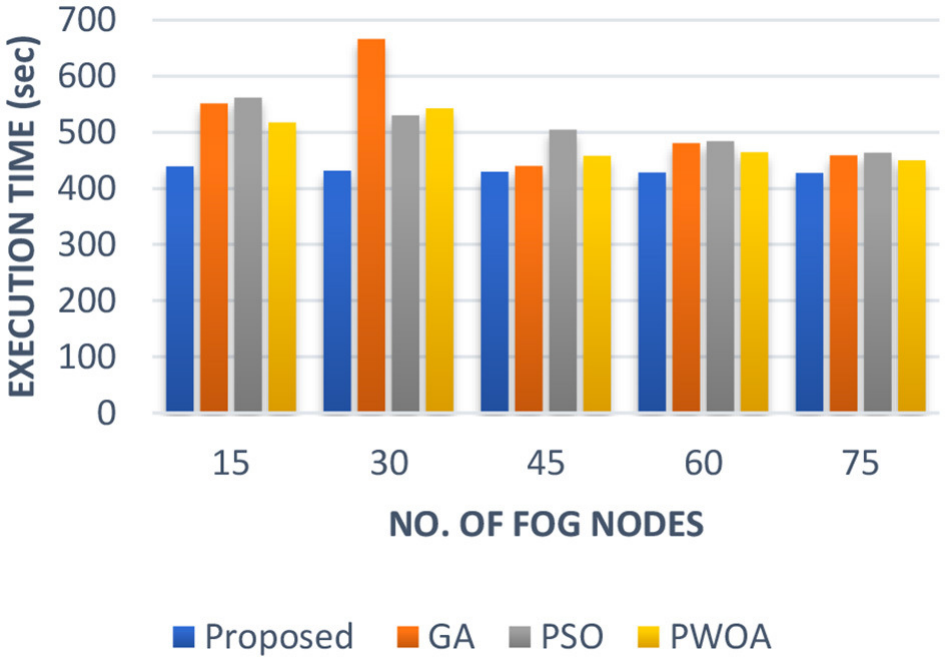
Comparison of Execution time (in second) of proposed Hybrid algorithm with conventional GA, Hybrid PWOA and PSO with different number of fog nodes.

**Figure 7:**
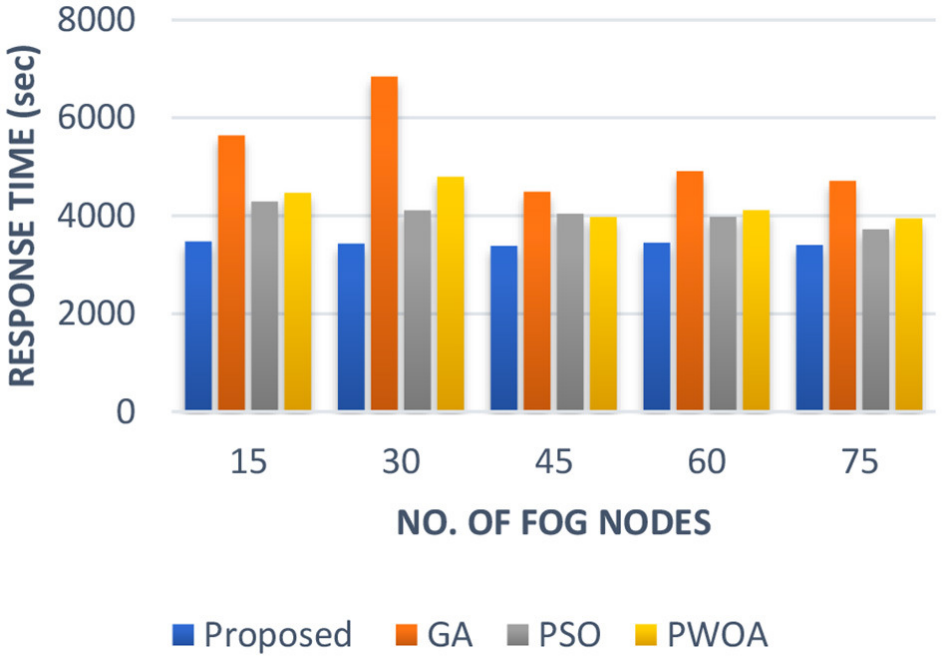
Comparison of Response time (in second) of proposed Hybrid algorithm with conventional GA, Hybrid PWOA and PSO with different number of fog nodes.

**Figure 8:**
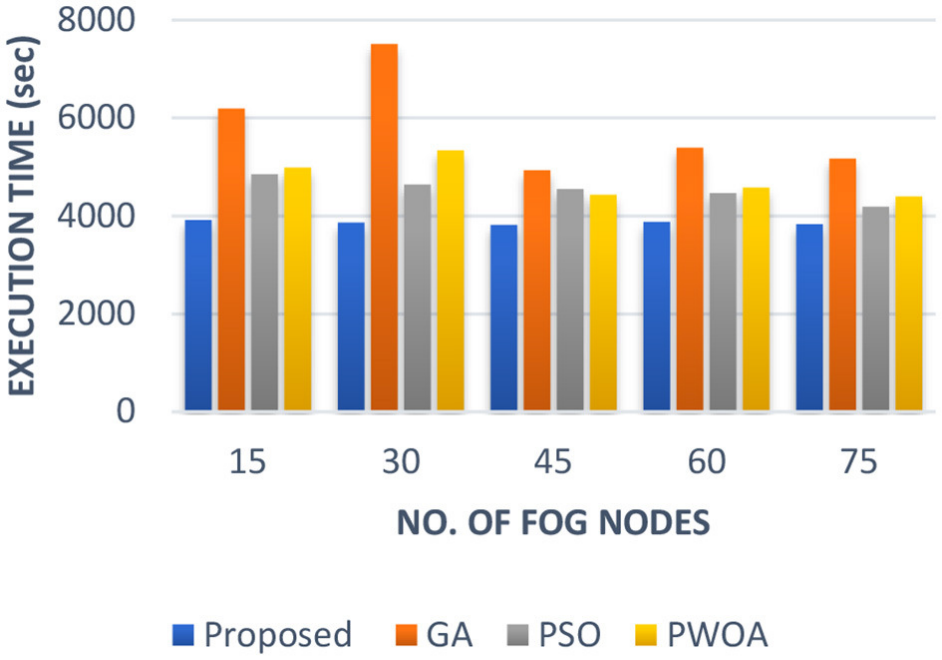
Comparison of Response time (in second) of proposed Hybrid algorithm with conventional GA, Hybrid PWOA and PSO with different number of fog nodes.

In Table 5 we have presented the outcomes of the proposed hybrid algorithm, GA algorithm, Hybrid PWOA algorithm and PSO algorithm in terms of execution time, response time and completion time when various fog nodes are given. Figure 6 indicates the proposed hybrid algorithm results improved the execution time by 84.87% when compared with GA algorithm, by 88.64% when compared with Hybrid PWOA and by 85.07% when compared with PSO algorithm. Figure 7 indicates the proposed hybrid algorithm results improved the response time by 65.92% when compared with GA algorithm, by 80.51% when compared with Hybrid PWOA and by 85.26% when compared with PSO algorithm. Figure 8 indicates the proposed hybrid algorithm results improved the completion time by 67.60% when compared with GA algorithm, by 81.34% when compared with Hybrid PWOA and by 85.23% when compared with PSO algorithm.

**Table 5:** The result of experiments number two.

**Algorithm**	**No. of fog nodes**	**Execution time (sec)**	**Response time (sec)**	**Completion time (sec)**
**Scenario One**				
GA-	15	439.33	3,476.44	3,915.77
GA		551.4	5,641.75	6,193.15
PSO		561.87	4,290.59	4,852.46
PWOA		517.53	4,469.59	4,987.12
**Scenario Two**				
GA-	30	431.52	3,433.28	3,864.79
GA		666.23	6,844.88	7,511.11
PSO		530.23	4,111.6	4,641.83
PWOA		542.66	4,796.59	5,339.25
**Scenario Three**				
GA-	45	429.69	3,386.39	3,816.08
GA		440.08	4,491.95	4,932.03
PSO		504.71	4,043.9	4,548.61
PWOA		458.16	3,974.08	4,432.24
**Scenario Four**				
GA-	60	428.39	3,450.72	3,879.11
GA		480.67	4,911.53	5,392.2
PSO		484.24	3,981.56	4,465.8
PWOA		464.43	4,114.60	4,579.03
**Scenario Five**				
GA-	75	427.57	3,403.79	3,831.36
GA		458.94	4,713.05	5,172
PSO		463.68	3,724.14	4,187.82
PWOA		450.06	3,946.99	4,397.05

Therefore, GA-PSO is the most efficient algorithm in terms of execution time, response time and completion time, followed by PSO, Hybrid PWOA and GA is the least efficient algorithm.

## 6 Conclusion

In this study, both the problems of task scheduling in fog computing environment and the solution to it are discussed by proposing a multi objective hybrid GA-PSO optimization algorithm. The experimental result shows that the proposed Hybrid GA-PSO algorithm-based task allocation give optimal results when compared with the GA, Hybrid PWOA and PSO algorithm in terms of both heterogeneity of fog nodes and number of data input respectively. Furthermore, the Hybrid GA-PSO technique does not always get trapped in the locally optimal solution due to GA's exploration skills with PSO's exploitation characteristics which increases the accuracy of the solution. We conducted two experiments and compare both algorithm outputs in terms of execution, response and completion time. In experiment one we fix the number of fog nodes and change number of data inputs and compare the results. We found that as the number of data input increases proposed Hybrid GA-PSO algorithm improves the execution time, response time, and completion time when compared with Hybrid PWOA, GA and PSO algorithm. In experiment two this time we fix the number of data input and change no of fog nodes in every scenario and compare the results. We found that as the number of fog nodes increases proposed Hybrid GA-PSO algorithm improves the execution time, response time, and completion time when compared with Hybrid PWOA, GA, and PSO algorithm. By combining the Genetic algorithm and particle swarm optimization algorithm, the performance of the multi objective task scheduling in fog computing environment is improved. In future, researchers can push the boundaries of hybrid optimization and develop even more powerful and versatile algorithms for solving complex real-world problems. The issue of uncertainty in allocating tasks to fog nodes must be resolved. Enhancing security and privacy protocols to handle sensitive large data in fog situations may need more refinement.

## Data availability statement

The original contributions presented in the study are included in the article/supplementary material, further inquiries can be directed to the corresponding author.

## Author contributions

MS: Writing – original draft, Writing – review & editing. RE: Formal analysis, Writing – review & editing. RQ: Formal analysis, Writing – review & editing.
